# Study on Influencing Factors of Micro and Small Enterprises’ Work Safety Behavior in Chinese High-Risk Industries

**DOI:** 10.3389/fpsyg.2022.880205

**Published:** 2022-05-16

**Authors:** Wen Li, Xitao Ni, Xiaolin Zuo, Suxia Liu, Qiang Mei

**Affiliations:** ^1^School of Management, Jiangsu University, Zhenjiang, China; ^2^China SME Project Research and Training Base, Zhenjiang, China

**Keywords:** work safety behavior, influencing factors, MSEs, high-risk industries, theory of planned behavior, complex social system

## Abstract

Due to the limited work safety resources and the poor awareness of work safety from business owners with absolute decision-making power, safety accidents frequently occur in Chinese micro and small enterprises (MSEs) in high-risk industries. This study identifies the influencing factors of work safety behavior from MSEs, government safety supervision departments, and work safety service agencies. Based on the theory of planned behavior (TPB), the mechanism model of work safety behavior is built from the aspects of behavior attitude, subjective norms, behavior control cognition, past behaviors, and risk awareness of the enterprise. Based on the interview with nearly 600 MSEs in the east of China over 6 months, the results show that the work safety awareness of the business owner determines the work safety lever of the enterprise, and the work safety behavior of MSEs is a passive restraint behavior. Our findings provide a new perspective on the formation of MSEs’ work safety behavior in high-risk industries.

## Introduction

Micro and small enterprises (MSEs) are the lifeblood in China’s economic and social development in recent years. It plays a pivotal role in China such as promoting economic growth, transformation, and upgrading; optimizing economic structure; expanding employment; increasing income; and improving people’s livelihood (Tremblay and Badri, 2018). Compared with foreign enterprises and state-owned large and medium-sized enterprises, MSEs, as the most dynamic group in China’s economy, are also the most vulnerable. High-risk MSEs have problems such as failure to implement work safety responsibilities, weak safety awareness, and confusion in safety management. In April 2018, during the transportation of explosives, a dangerous transport vehicle belonging to Gushi County Chemical Co., Ltd., Henan in Yuehe Town, Zhen’an County, Shanxi Province, exploded due to the transporter’s unsafe operation, causing seven deaths and nineteen injuries. To minimize the operating costs, the plant infrastructure of the MSEs is primitive with hidden dangers. Production technology is backward, and the work environment is adverse. It can be seen that the MSEs have problems of “lack management” or “unable to manage” (Olsen and Hasle, 2015), so serious accidents occur frequently. According to statistics in China, in recent years, more than 70% of major accidents are concentrated in MSEs in high-risk industries, especially coal production, transportation, and construction engineering. The frequent occurrence of major accidents in high-risk industries has not been effectively curbed. The work safety situation is still grim, and there are still a large number of accidents.

Since MSEs in high-risk industries are small in scale, difficult in financing, difficult in employment, and high in operating costs, it is difficult for enterprises to realize work safety with limited profit space. Business owners have a fluke mentality for safety accidents, lack awareness of work safety, and lack pressure and initiative for work safety. At the same time, “Accidents Occurring-Inspection and Regulation-Closing” has become the routine for the work safety supervision department to deal with work safety accidents (Mei et al., 2017). The government focuses on the time and quantity of the work safety of MSEs. The insufficient supervision or partial supervision failure by the government supervision department causes it difficult to curb the accidents from the source; in addition, work safety service agencies are more concerned about how to satisfy the standards more easily. Due to the lack of supervision and constraint, these agencies even collude with enterprises to implement the standards. It is too formalistic for MSEs in high-risk industries, government work safety supervision departments, and work safety service agencies to implement work safety behaviors.

To explore the influence factors of the work safety behaviors, the enterprise business owners are taken as the research object to fully explore the mechanism of the work safety behavior of MSEs in high-risk industries. In this study, more than 600 MSEs in the east of China are selected. These enterprises are mainly involved in high-risk industries such as constructional engineering, transportation, hazardous material production and storage, non-coal mining, machinery manufacturing, fireworks production, and metallurgy. It is expected to demonstrate the internal influence mechanism of the enterprise behavior attitude, subjective norms, perceptual control behavior, enterprise past behavior, and risk preference on the work safety behavior of MSEs in Chinese high-risk industries.

## Literature Review

Recently, a study on the work safety behavior of MSEs in high-risk industries focuses more on the supervision of enterprises, the safety evaluation of enterprise accidents in high-risk industries, and the work safety entrust. The work safety supervision of high-risk MSEs is complex, and the spontaneous evolution modes of supervision strategies are analyzed. It is necessary for high-risk enterprises to obtain work safety licenses to engage in production, and one of the conditions for obtaining a work safety license is to pass the safety evaluation (Zhang et al., 2017). For the convenience of subsequent research, the work safety behaviors are divided into forward and reverse aspects (Liu et al., 2015). As the basic organizational form and entities of economic activities, enterprises have “organizational behaviors” in safety activities (Daft, 2012), i.e., “the safety behavior of the enterprises.” The behavior directly affects the safety of people and the things, and it directly affects the safety level of the enterprise. The work safety behavior of an enterprise mainly reflects the safety decisions by managers or management who have budget decision-making power, resource allocation power, and job placement priority (Liu et al., 2012). Combined with the decision made by the MSE owners with decision-making power, the work safety behaviors of MSEs are divided into two dimensions, namely, work safety behavior and work unsafe behavior. Based on the current situation, work safety behaviors can be realized by purchasing work safety services or implementing the safety standard independently. Thus, work safety behaviors are divided into service-oriented work safety behavior and independent work safety behavior.

The theory of planned behavior is a mature theoretical research model developed on the basis of the theory of reasoned action. Ajzen put forward the theory of reasoned action based on the theory of multi-attribute attitudes firstly (Ajzen, 1985), and it was continuously improved to form the theory of planned behavior (TPB). There are many empirical studies on predicting and explaining individual behavior through the theory of planned behavior, but there are few studies on explaining the behavior decisions of enterprises (organizations) by using the theory of planned behavior. The business owners (top management) of MSEs make decisions on the work safety behavior of the enterprise. Combined with the work safety characteristics of MSEs in high-risk industries, it is feasible to study the mechanism of work safety behavior of MSEs based on the theory of planned behavior. Based on the theory of planned behavior, the influence of three traditional variables on behavioral intention and behavior is considered when the work safety behavior of MSEs is studied. Among them, behavior attitude mainly reflects the cognition of business owners with decision-making power to the enterprise work safety, which involves the enterprise strategic planning, operation management, and specific work safety behaviors (Mullen et al., 2017). The subjective norms originate from external constraints on enterprises, and it involves two aspects, namely, constraints and norms. The perceptual behavior control mainly reflects the resource support that is beneficial to the work safety behavior of enterprises.

## Identification of Influencing Factors

### Analysis of Stakeholders on Work Safety Behavior of Micro and Small Enterprises

**FIGURE 1:**
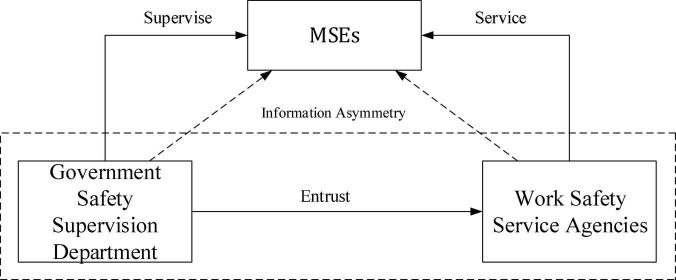
Operating system structure of the work safety behaviors of micro and small enterprises (MSEs).

The work safety behavior of MSEs in high-risk industries is mainly influenced by the stakeholders such as MSEs, government safety supervision department, work safety service agencies, and external environment, as shown in Figure 1.

(1) Influencing factors of work safety behavior of MSEs:

Due to a variety of subjective and objective factors such as the research field, there are large differences in the analysis dimensions of work safety behavior. The enterprise work safety behavior includes management commitment, safety training, employee participation, safety communication and feedback, safety regulations, and safety improvement strategies (Vinodkumar and Bhasi, 2010). From the perspective of safety atmosphere, it can be divided into management values, management practices, communication, and employees’ involvement in safety and health in the workplace (Neal et al., 2000). Based on the factor analysis method, the work safety behavior can be divided into seven dimensions, including manager safety, work safety, colleague safety, safety management, safety training, safety regulations, special safety training, and work pressure (Liu et al., 2009). In China, there are many methods to divide the work safety behavior, including classification based on the nature of safety investment and classification based on the function of safety investment (Chen et al., 2004; Lu and Mei, 2008).

In high-risk industries, Cooper studied the impact of managers’ participation in enterprise safety on employee work safety behavior, and the results showed that the safety participation of enterprise managers greatly influences the unsafe behavior of employees of the enterprise. The manager with a higher position can influence the behavior of the employees more (Cooper, 2006). The senior managers influence the behavior of the lower managers and then the behavior of the entire enterprise. Hence, the business owners (top managers) of MSEs play a leading role in the work safety behavior decision of MSEs.

In this study, the influencing factors of work safety behaviors of MSEs are divided into the following four aspects: the cognitive factors of business owners, i.e., the impact of managers on work safety awareness; the organizational factors, i.e., the impact of industry characteristics and organizational structure of MSEs; the economic factors, i.e., the impact of work safety behaviors of MSEs on cost accounting and benefit analysis; the risk factors, i.e., risk identification of MSEs and its impact on consciousness behavior.

The cognitive factors refer to the behavior attitudes of MSEs’ owners toward work safety behaviors. It involves the integration of enterprise strategic positioning, emphasis, and positive measures. The managers of MSEs control the decision-making power and management power of the enterprise. The implementation and decision-making of the enterprise work safety behavior depend on the safety awareness of the business owner, and it guides the enterprise work safety behavior. Business owners with higher safety awareness implement work safety behaviors actively and positively, while business owners with lower safety awareness often passively or even do not implement work safety behaviors. It can be seen that the attention of micro and small enterprise owners give to work safety behavior and their awareness of taking the initiative to implement work safety behavior directly affect the work safety behavior of MSEs.

The organizational factors refer to factors such as whether an enterprise has established a special safety management organization, the operation mechanism, and the authority of these organizations. According to the “Law on Work Safety” in China, special safety management agencies or full-time safety management personnel are required in enterprises in high-risk industries, including mines, construction organization, and units that produce, operate, and store hazardous materials. Through the preliminary investigation of MSEs in high-risk industries in Jiangsu Province, it is found that the work safety management department is set in 32% of MSEs; in 65% of MSEs, work safety agency functions are supervised by other departments within the enterprise; and there is no work safety agency in a few individual operation enterprises. In addition, there is no full-time or part-time safety management personnel in most MSEs, and some special operations personnel do not have certificates. The work safety of many MSEs without safety management institutions is basically neglected. Therefore, the imperfect work safety management organization of enterprises and the lacking full-time and part-time safety management personnel are regarded as organizational factors affecting the work safety behavior of MSEs.

The economic factors are one of the important influencing factors restricting the work safety behavior of MSEs. This part is studied from the perspective of the safe investment behavior of business owners. It is believed by enterprises that safety investment only increases the cost of the enterprise and there are no economic profits. From the reality of MSEs in high-risk industries, all expenses related to enterprise safety are not calculated and managed separately but included in various departments or corresponding expenses as production costs and management expenses (Lu and Mei, 2010). The lack of a safety cost accounting system limits the ideas of cost control for MSEs. Without the objective understanding of safety input-output-benefit, MSEs’ judgment on safety benefits and their future safety investment are affected. In the enterprise operation, the enterprise’s safety awareness or the severity of accidents is evaluated from whether the enterprise actively pays its employees’ injury insurance. Lax enforcement causes lag, potential, the externality of safety benefits perceived by the enterprises, leading to the gap between the cost and benefit and the actual value of safety investment behaviors, which causes the improper safety investment of the enterprises.

The risk factors are the identification and attitude of micro and small business owners to risk and its impact on the risk-taking behavior of employees. In the production of enterprises, combined with the industry characteristics of enterprises, the new “Law on Work Safety” points out that the warning signs of major safety sources should be highly visible, personnel for special operations are required, and operation should be provided regularly and continuously. Micro and small business owners believe that risks may not cause harm to employees, they ignore the danger of safety accidents, and they put fluke psychology about the risks in the production. Business owners do not value risks, resulting in unsafe production behaviors of employees.

(2) Regulatory constraints of the government safety supervision department:

Based on the economic development, the work safety system in China has been constantly adjusted, and a new supervision and management system with “unified leadership by the government, department supervision in accordance with the law, enterprise taking full responsibility, public participation in supervision, and broad support from the whole society” has been formed (Li et al., 2006). However, due to the particularity of MSEs, they are at a disadvantage in the process of being supervised by the government in work safety. Most of the MSEs are located in townships, but there are no safety supervision institutions and personnel set in most of the townships. Even if there are safety supervision institutions and personnel, they have no law enforcement power, leaving gaps in safety supervision. The existing government management modes are unsuitable for the development. On the one hand, many government departments still follow the management modes under the planned economic system. They intervene directly in markets, instead of is making guidance, and inspect and charge for the market, instead of providing supervision services, without effective means of safety supervision; on the other hand, insufficient safety supervision. Many MSEs are in a state of safety supervision gap or supervision failure. From the perspective of safety supervision, there are problems in the work safety regulation of MSEs in China, such as poor work safety-related information and low effectiveness of work safety regulation. The work safety standards and the imperfect socialized service system for work safety behavior of MSEs have led to excessive regulation pressure of the government (Mei and Liu, 2010).

(3) Supporting from work safety service agencies:

Work safety service agencies are generated in the market economic system. Entrusted by government departments, it engages in specialized technical service activities such as work safety evaluation, certification, monitoring, inspection, and consulting services in accordance with *Work Safety Law*. With the gradual improvement and development of the market economic system, the work safety service organization can effectively solve the defects of MSEs’ work safety such as insufficient human resources, incompatible safety facilities, and extensive work safety management. The evaluation, certification, qualification of occupational safety and health of employees, and safety training are finished by the work safety service organizations, so they are required to further improve their service levels and quality (Zhong et al., 2014). The government provides preferential policies for MSEs through certification, establishing or funding work safety service organizations, and encourages service agencies to provide MSEs with safety technical support, safety training, consulting, testing, and system demonstration. In addition, the government will also supervise the training effects and safety evaluation reliability of work safety service organizations, conduct tracking to ensure the service level of service organizations, and ban the unqualified service organizations.

(4) Other subjects of the external environment:

Research on the work safety behavior of MSEs does not only involve enterprises, government safety supervision departments, or work safety service agencies. Other influencing factors should be considered, such as the employee demands, the demonstration role of the work safety behavior of neighboring enterprises, the public opinion pressure, and the work safety constraints and regulations of upstream and downstream enterprises in the supply chain.

### Influencing Factors of Work Safety Behavior of Micro and Small Enterprises

Influencing factors of the work safety behavior of MSEs in high-risk industries are analyzed from internal and external dimensions of enterprise to form a classification system, as shown in Table 1.

**TABLE 1:** Classification of influencing factors of work safety behavior of micro and small enterprises (MSEs).

Category	Influencing factors	Specific description
Internal factors of enterprises	Cognition factor of business owners	The attention of business owners to work safety, business owners’ willingness to invest in safety, or the role of work safety in strategic planning
	Organization factors	The industry characteristics (whether it is a high-risk industry), the setting and perfection of work safety management organization, and the configuration of full-time (or part-time) safety management personnel, etc.
	Economic factors	The accounting of safety cost in cost accounting (classification), the proportion of safety cost in the total cost, whether the safety cost brings direct benefits (or indirect benefits) to the enterprise, the proportion of the safety cost, etc.
	Risk factors	The ability to identify the existing hazards of the enterprise, the ability to regulation and restraint of major hazards, and the business owners’ awareness of risk-taking
Enterprise stakeholders	Government regulation	Setting of the safety department and safety management personnel in local government are efforts in supervising, whether there are regular safety inspections. The implementation of the administrative license for work safety, whether effective potential danger rectification and tracking procedures are established.
	Safety demands from employees	Whether the enterprise conducts safety education and training for employees, whether employees can identify hazards at work, whether the enterprise provides all employees with necessary protective equipment and conduct regular inspection and maintenance, and whether the enterprise purchase injury insurance for employees
	Safety services and technical support of service agencies	Whether the enterprise can easily obtain the safety services of the safety intermediary organizations (such as training methods, standardization of technical services, etc.), whether the enterprise can implement the rectification opinions, whether the enterprise has formed a long-term cooperation with the organization, etc.
	Public opinion restraint	The safety demands of the public, whether the pressure of public opinion has a positive effect on the work safety behavior of MSEs, etc.
	Work safety behaviors of neighboring enterprises	The work safety strategy planning, and demonstration role of work safety behaviors of neighboring enterprises, etc.
	Safety standards of upstream and downstream enterprises in the supply chain	Whether suppliers, distributors, and consumers have signed information sharing contracts to control the binding force of enterprise work safety and safety protection, and there is a unified information system platform to achieve enterprise credit evaluation, etc.

Based on the classic theory of planned behavior theory, considering the impact of past behaviors on the current work safety behavior of enterprises and the risk characteristics of high-risk industries, two exogenous variables, behavior habits, and risk awareness are introduced. Combined with these two factors, traditional behavior attitudes, subjective norms, perceptual behavior control, and behavioral habits affect the work safety behavior through the work safety behavioral intention.

Behavior attitude mainly reflects the cognitive factors of enterprise owners with decision-making power on work safety behavior of MSEs, involving enterprise strategic planning, operation management, and specific work safety behavior. Subjective norms are derived from external constraints on MSEs, which generally involve hard norms and soft constraints. Perceptual behavior control mainly shows the favorable resource for MSEs’ work safety behavior, including internal work safety resources, capital and policy support provided by the government safety supervision department, and technical support provided by work safety service agencies. Behavior habits mainly consider the impact of the past behavior of enterprises and the behavior of neighbor enterprises on the work safety behavior of MSEs. Risk awareness reflects the risk characteristics of high-risk industries, including the identification of existing hazard sources, the standardized operation awareness of major hazard sources, and employees’ conformity psychology. The connotation of these exogenous variables and the influencing factors based on stakeholders are studied, and the corresponding relationship is formed, as shown in Table 2.

**TABLE 2:** The correspondence relationship between the exogenous variables and the influencing factors of MSEs’ work safety behaviors in high-risk industries.

Influencing factors in the theory of planned behavior	Influencing factors of MSEs’ work safety behavior
Behavior attitude	Business owners’ awareness of work safety, strategic positioning of work safety issues in the enterprise, and the importance of work safety in the operation and management of the enterprise
Subjective norms	Government supervision, employee safety demands, implementation of rectification opinions in service agencies, public opinion constraints, industry norms constraints, the definition of safety standards of upstream and downstream companies in the supply chain
Perceptual behavior control	Constraints of the existing work safety resources of the enterprise (the establishment of enterprise work safety management department, the configuration of work safety management personnel), support of work safety policy in government, work safety technical support from service agencies
Behavioral habits	The past work safety behaviors of enterprises, the work safety behavior norms of neighboring enterprises
Risk awareness	The ability to identify the existing hazards of the enterprise, the ability to regulate and restrain the major hazards, and the business owners’ awareness of risk-taking attempts

## Hypotheses

(1) Relationship hypothesis between behavior attitude (A) and behavioral intention (I) of MSEs in high-risk industries:

Ajzen tested the theory of planned behavior by behaviors such as losing weight, the attendance rate of college courses, and getting an “A” in college examinations. The research showed that the prediction of behavioral intention based on three independent variables: attitude, subjective norms and perceived behavior control is successful (Ajzen, 1991). The drivers’ driving violation behavior was studied, and it was found that the motor vehicle driver’s attitude toward driving violation behavior can be predicted through the mediating effect of behavioral intention (Ding, 2006). Pedestrians’ motivations for violating traffic rules were studied, and self-assessment measurements were conducted on 146 pedestrians. The results showed that behavior attitude is related to behavioral intention, and behavior attitude, subjective norms, and perceptual behavior control are also related to each other (Diaz, 2002). Based on the theory of planned behavior, Lu studied the uncivilized behavior of tourists and found that the attitude of tourists in an unusual environment is the most influential factor of the uncivilized behavioral intention (Lu et al., 2019).

It can be seen that the influence of the behavior attitude, subjective norms, and perceptual behavior control on behavioral intention has been confirmed to varying degrees. Scholars at home and abroad have widely confirmed that behavior attitudes positively affect behavioral intentions (Hammami et al., 2013). Some scholars do not support the relationship between the two clearly, but they do not deny the direct connection between behavior attitude and behavioral intention.

As a durable and stable estimable psychological composition, behavior attitude can influence and predict the occurrence of behavior to a certain extent. Is the behavior taken by micro and small business owners determined by the attitude in high-risk industries? Therefore, the following hypothesis is proposed:

**H1:** Behavior attitude (A) positively affects the behavioral intention (I).

(2) Relationship hypothesis among the subjective norms (SN), behavior attitudes (A), and behavioral intention (I) of MSEs in high-risk industries:

There is a very significant correlation between subjective norms and behavioral intentions (Ajzen et al., 2011). Initially, subjective norms were parallel to behavior attitudes in the theory of reasoned action, and they influenced behavioral intentions as independent variables. Combined with the actual situation, it can be determined that subjective norms have a positive or negative influence on behavioral intentions (Huang et al., 2013). Subjective norms mainly reflect hard norms from government supervision and soft norms from employee safety demands, industry associations, public opinion, and upstream and downstream enterprises in the supply chain. The following hypothesis is proposed:

**H2:** subjective norms (SN) have a positive effect on behavioral intentions (I).

First, in the theory of reasoned action, subjective norms and behavior attitudes were in a parallel state, and they affected behavior intention as exogenous variables (Smith et al., 2007). The relationship is verified between subjective norms and behavior attitude by using empirical data. Moreover, subjective norms influence behavioral intentions. Lan found in empirical research that subjective norm does not affect behavioral intentions directly, but it influences behavioral intentions by acting on behavior attitudes (Lan and Zhu, 2009). It is necessary to analyze whether subjective norms affect behavioral intention indirectly through behavior attitudes of MSEs in high-risk industries. Thus, the following hypothesis is proposed:

**H3:** behavior attitude (A) plays a mediating role in the influence of subjective norms (SN) on behavioral intentions (I).

(3) Relationship hypothesis between perceptual behavior control (PBC) and the behavioral intention (I) of MSEs in high-risk industries:

The factors that cannot be explained by behavior attitudes and subjective norms are included in perceptual behavior control variables by many scholars. Based on TPB theory, Zhu constructed a configuration analysis model of internal employee entrepreneurial behavior (Zhu and Guo, 2020). The empirical results showed that the joint action of perceptual behavior control, behavior attitude, and subjective norms has multiple causalities and equivalence characteristics on internal employee entrepreneurial behavioral intention. The direct relationship between perceptual behavior control and behavior intention has been confirmed by empirical research of scholars. In this paper, the perceptual behavior control is integrated into the formation of favorable resource support for the work safety behavior of MSEs in high-risk industries, including the internal enterprise resources, the technical support and supervision management of the service organization, the policy support provided by the government, etc. It is necessary to analyze whether these resources have a positive effect on the formation of work safety behavior of MSEs in high-risk industries. The following hypothesis is proposed:

**H4:** Perceptual behavior control (PBC) positively affects the behavioral intention (I) of work safety.

(4) Relationship hypothesis among behavior attitude (A), behavior habit (BH), and behavioral intention (I) of MSEs in high-risk industries:

Many scholars believe that people always act in a certain way. Therefore, as long as this behavior has appeared, it is highly likely that the behavior will be repeated. “More than 90% of things people do every day are almost completely compliant with customary procedures,” which is behavior habits. Past behavior improves the ability to predict behavior as a part of behavior control cognition in the theory of planned behavior (Norman and Cooper, 2011). Behavioral habits cannot be effectively included in the perceptual behavior control but enter into the model as an independent component. Behavioral habits influence behavioral intentions (Bagozzi and Yi, 1988). When the behavior is not perceived by the subject or when the behavior is in an unstable state, the behavior subject must consciously decide whether or not to perform the action. In this way, behavior is likely to affect behavioral intentions.

In addition, many other studies have confirmed that past behavior directly affects behavioral intention or behavior, not through attitude. For example, when the work safety behaviors are studied, the past behaviors or the influence of past experience are considered (Zwetsloot et al., 2011). If people tend to be accustomed to illegal actions, there may be a significant correlation between the past and future behaviors. It is believed that the previous violation did not cause an accident, so the behavior will not cause an accident. Therefore, the following hypotheses are proposed:

**H5:** Behavior habit (BH) significantly influences behavioral intention (I).

**H6:** Behavior attitude (A) plays a mediating role in the influence of behavior habit (BH) on behavioral intention (I).

(5) Relationship hypothesis between work safety risk awareness (RC) and behavior intention (I) of MSEs in high-risk industries:

The risk-related field was first proposed in the prospect theory. The theory proposed that people’s decisions about gains and losses are asymmetric. Most people avoid risk when facing gains, but they prefer risk when facing loss. Based on the theory, a lot of studies on risk propensity were conducted. By analyzing the relationship between personality characteristics and risky behaviors, Niskanen found that there is a close positive correlation between people with high-risk preferences and risky behaviors (Niskanen et al., 2012). For users with high-risk tendencies, even the best safety signs are unlikely to be followed.

Larsson (2008) found that people with high-risk preferences are unwilling to follow safe behaviors. People with high-risk preferences do not adopt safe behaviors but an easier way during operation (Martínez-Córcoles et al., 2012). Combined with the hazard identification characteristics of MSEs in high-risk industries, individual awareness of the hazards is introduced, and the authors attempted to introduce the work safety risk awareness variable into the TPB. The following hypothesis is proposed:

**H7:** Risk consciousness (RP) significantly influences behavioral intention (I).

(6) Relationship hypothesis between work safety behavioral intention (I) and behavior (B) of MSEs in high-risk industries:

Ajzen believed that after the individual recognizes the behavior, with sufficient resources and opportunity, people will have behavior expectations and put them into action (Cordano and Frieze, 2000). Therefore, in a specific situation, the work safety behavioral intention produces a behavioral affecting the result after a certain behavioral expectation, which is an intermediary variable that causes work safety behavior. Hence, based on the definition of enterprise work safety planning and expected work safety resource input, the authors analyzed the significant influence of behavioral intention on work safety behavior of MSEs in high-risk industries and proposed the following hypothesis:

**H8:** Behavioral intention (I) influences work safety behavior (B) significantly.

## Construction of Decision-Making Theoretical Models

**FIGURE 2:**
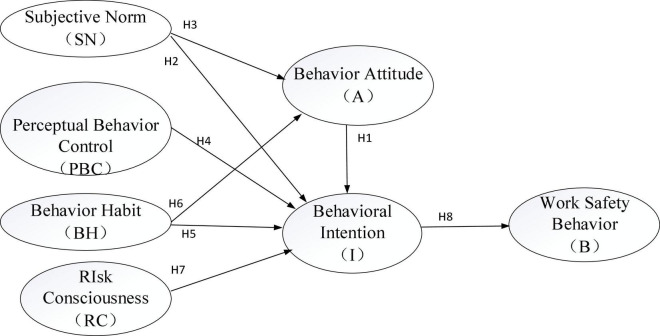
A conceptual model for the influence mechanism of MSEs’ work safety behavior in high-risk industries.

Based on the above theoretical hypotheses, combined with the theory of planned behavior and the research conclusions by scholars, the influence of behavior attitudes, subjective norms, and behavior control cognition on behavioral intentions are studied. Meanwhile, risk preference and behavior habits are introduced to the model, and it is expected to understand whether two variables can significantly affect the work safety behavior and intention of MSEs. The behavioral intention reflects an individual’s willingness to engage in a certain behavior, which is the most important predictor of behavior and directly determines work safety behavior. Therefore, the conceptual model of work safety behavior of MSEs in high-risk industries is constructed, as shown in Figure 2.

## Research Design

Based on authoritative theoretical viewpoints and scales used in the existing empirical research, combined with the actual situation of work safety behavior of MSEs in high-risk industries in China, an initial scale on work safety behavior of MSEs in high-risk industries is formed through expert interviews and consultations. An initial questionnaire test was conducted on 80 MSEs in high-risk industries in Jingkou District and Runzhou District of Zhenjiang City, Jiangsu Province. The high-risk industries include machinery manufacturing, chemical industry, metallurgy, transportation, and construction engineering. Finally, a formal questionnaire is obtained, as shown in Supplementary Appendix.

From December 2019 to June 2020, a questionnaire survey was conducted for MSEs in high-risk industries in the east of China, involving seven high-risk industries, including machinery manufacturing, constructional engineering, production and storage of hazardous material, transportation, non-coal mining, fireworks production, and metallurgy. A total of 600 questionnaires were issued, and 512 questionnaires were recovered. Among them, 445 valid questionnaires were received with a recovery rate of 74.17%.

Most of the MSEs surveyed are private enterprises, and small parts are companies with limited liability or self-employed. The business owners in high-risk industries are surveyed. In total, 36.4% of business owners have a college or bachelor’s degree, and 53.2% of business owners have a high school degree. The enterprise information in high-risk industries surveyed is shown in Table 3.

**TABLE 3:** Industry distribution of the enterprises.

Industry	Number	Proportion (%)
Machinery manufacturing	162	36.4
Constructional engineering	72	16.19
Non-coal mining	19	4.27
Hazardous material production and storage	42	9.44
Transportation	17	3.82
Fireworks production	16	3.6
Metallurgy	117	26.28

### Data Analysis

#### Reliability Test of the Scale

The Cronbach’s α coefficient for reliability testing is a new method that is widely used. The higher the coefficient, the better the internal consistency. It is generally considered that the internal consistency is acceptable when the coefficient is greater than 0.7. Table 4 shows the reliability of the scale in SPSS 22. The results show that the Cronbach’s α coefficient of the seven variables are all greater than 0.7, showing a good internal consistency reliability.

**TABLE 4:** Reliability test of the scale involved in the model.

Dimension	Item	Computed Cronbach’s α
Behavior attitude (A)	7	0.849
Subjective norm (SN)	11	0.881
Perceptual behavior control (PBC)	8	0.821
Behavior habit (BH)	10	0.879
Risk consciousness (RC)	6	0.725
Behavioral intention (I)	5	0.858
Work safety behavior (B)	15	0.933

#### Validity Analysis of the Scale

Validity analysis is generally divided into content validity, criterion validity, and construct validity. Among them, the construct validity is finished through exploratory factor analysis (EFA) and confirmatory factor analysis (CFA). EFA is used to explore the underlying structure of the observed variables. CFA is used to test the existence of latent variables and factor structure. Some scholars believe that if the scale designed is a mature scale, it can be tested by CFA. For scales constructed based on specific theories and actual situations, CFA and EFA are both needed to verify the construct validity of the questionnaire. For EFA, principal component analysis and the maximum variance method are used to extract eigenvalues and primary common factors (Wu, 2010). The items whose factor loading is less than 0.45 are eliminated. If it exceeds 0.7, the effect is the best. For CFA, the goodness-of-fit test and convergence effect test are conducted.

There are seven variables in the study of the work safety behavior of MSEs, including five exogenous variables, one intermediate variable, and one endogenous variable. The total number of questions for all variables is up to 63. It is difficult to carry out the validity test as a whole. This study tests the validity for each variable separately. Too many tests are finished in AMOS 24, and the test process is roughly similar. Here, the construct validity test of perceptual behavior control (PBC) is finished, and the test results are listed in Table 5.

**TABLE 5:** EFA results of the perceived behavioral control.

Perceived behavioral control	Item	Factor loading			Eigen value (Explained variance)
		Common factor 1	Common factor 2	Common factor 3	
Internal source of the enterprise	PBC1	0.886			2.953 (32.672%)
	PBC2	0.898			
	PBC3	0.899			
	PBC4	0.888			
Support of service organizations	PBC5		0.786		1.978 (21.678%)
	PBC6		0.812		
	PBC7		0.811		
Government support	PBC8			0.764	1.532 (18.352%)
	PBC9			0.801	
Total explained variance	72.702%				

There are 9 questions on perceptual behavior control, as shown in Table 6. This variable can explain 72.702% of the variance. The factor analysis results further show that common factor 1 can explain items PBC1-PBC4 better, common factor 2 can explain items PBC5-PBC7 better, and common factor 3 can explain items PBC8-PBC9 better, showing good convergence of the variable. Therefore, on the basis of perceptual behavioral control, three common factors are extracted, that is, three new variables are formed, which are named as internal resources of the enterprise (PBCCF1), support of service organization (PBCCF2), and government support (PBCCF3). These three common factors test the problems of the internal resources of the enterprise, the support of safety service organizations, and the policy support provided by the government.

**TABLE 6:** Convergence validity test results of the perceived behavioral control in CFA.

Perceived behavioral control	Item	Normalized factor loading	AVE	CR
Internal source of the enterprise	PBC1	0.886	0.797	0.9401
	PBC2	0.898		
	PBC3	0.899		
	PBC4	0.888		
Support of service organizations	PBC5	0.786	0.645	0.8449
	PBC6	0.812		
	PBC7	0.811		
Government support	PBC8	0.764	0.6126	0.7597
	PBC9	0.801		

Then, AMOS 24, AVE, and CR calculation tools are used to conduct the convergence validity of the scale. The results are shown in Table 6.

Among them, the AVE values are all greater than 0.5, and the CR values are all greater than 0.7, showing good convergence. The convergent validity is tested by perceptual behavioral control.

The results of the construct validity test show that behavior attitude (A), subjective norm (SN), risk consciousness (RC), and behavioral intention (I) identify a common factor; three common factors are identified by perceptual behavioral control (PBC), and they are internal resources of enterprise (PBCCF1), support of service organization (PBCCF2), and government support (PBCCF3); two common factors are identified by the behavioral habits, and they are the past behavior of the enterprise (BHCF1) and the code of conduct of neighboring enterprises (BHCF2); and three common factors are identified by work safety behaviors, and they are safety management (BCF1), safety training (BCF2), and safety prevention (BCF3). All of them pass tests and show good construct validity.

### Model Fitting Test and Hypothesis Test

(1) Model fitting test:

The maximum likelihood method is used to estimate the model in AMOS 24. At the first fitting stage of the structural equation, as shown in Table 7, compared with the listed parameters of the fitting indicators, the ratio of chi-square degrees of freedom is 3.09 and the RMSEA is 0.0820, which do not meet the standard. Other parameter values meet the standard. The model needs to be further optimized and adjusted (Browne and Cudeck, 1992; Rong, 2009).

**TABLE 7:** Tests of goodness fit for the structural equations.

Index value	Computed X^2^	X^2^/*df*	GFI	RMSEA	AGFI	IFI	TLI	CFI
Amos test	1872.3	3.09	0.811	0.0820	0.918	0.901	0.912	0.907
Reference value	—	<3	>0.8	<0.08	>0.9	>0.90	>0.90	>0.90

A model modification tool (Modification Indices) is provided by AMOS 24, and it can be used to adjust the goodness of fit. Because the influence of the “behavior habit” on “behavioral intention” (β = 0.133, *T*-value = 0.743) is not significant. After the hypothesis H5 is excluded, the parameter values of the fitting indices increase slightly, and the modified index is shown in Table 8.

**TABLE 8:** Evaluation of modified model fitting.

Index value	Computed X^2^	X^2^/*df*	GFI	RMSEA	AGFI	IFI	TLI	CFI
Amos test	1275.96	2.058	0.898	0.071	0.821	0.921	0.968	0.956
Reference value	—	<3	>0.8	<0.08	>0.8	>0.90	>0.90	>0.90

The degree of freedom ratio of χ^2^/*df* value is less than 3, the RMSEA is less than 0.08, and the values of IFI, CFI, GFI, and AGFI are ideal. The model fits well.

(2) The mediating effect test of behavior attitudes:

Bootstrap is the optimal method for mediating effect test, which is widely used in various fields. In this method, the research sample is taken as the sampling population, and new sample data are generated by repeatedly sampling the overall sample. The average value of the sampled parameter is the final estimation result to obtain a result that is highly accurate and reliable (Wen and Ye, 2014). Hypothesis 5 (H5) is excluded, and subjective norms affecting behavioral intentions indirectly through behavior attitudes are presented, so the mediating effect of safety attitudes is studied. The Bootstrap is used, the confidence interval is set to 95%, and the sample is run for 2000 times (Wen et al., 2010). The mediation effect is obtained in SPSS 22, as shown in Table 9.

**TABLE 9:** Results of the mediating effect based on Bootstrap.

Path	Effect	Effect size	Squared error	95% confidence interval	
				Lower limit	Upper limit
Subjective norms→Safety	Indirect effect	0.2415	0.0453	0.1534	0.3321
Attitude→Safety intention	Direct effect	0.51	0.0325	0.6901	0.8153

First, the indirect effect value in the 95% confidence interval of the mediating effect is observed; it ranges from 0.1534 to 0.3321, and 0 is not included. The effect size is 0.2415, indicating that the mediating effect is significant. After the significance of mediating effect is tested, the direct influence test of the independent variable on the dependent variable is significant. Checked the direct effect value in the 95% confidence interval of the mediating effect, the value is in the interval (0.6901–0.8153), and 0 is not included. The effect size is 0.51. Therefore, the direct effect of the independent variable on the dependent variable is also significant. The mediating test of safety attitudes indicates that safety attitude plays a partial mediating effect, implying that subjective norms partially affect behavioral intentions directly and affect behavioral intentions through behavior attitudes indirectly.

**FIGURE 3:**
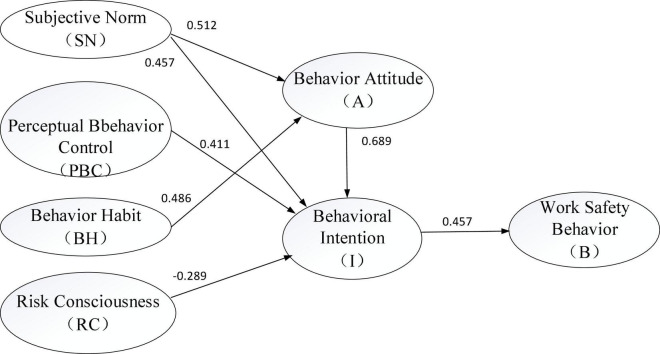
Influence mechanism model of the work safety behaviors of MSEs based on the theory of planned behavior in high-risk industries.

(3) Hypothesis test:

Combined with the results of the optimized fitting and mediating effect test, the influence mechanism model of the work safety behavior of MSEs based on the theory of planned behavior is finally established (Figure 2). This model not only shows the direct influence of behavior attitudes, subjective norms, perceptual behavioral control, and risk tendency on behavioral intentions, but it also shows the indirect influence of subjective norms and past behaviors on behavioral intentions. It can be found that subjective norms have the greatest influence on behavioral intention through the mediating effect of behavior attitude, and the influence coefficient is 0.81 (0.81 = SN→path coefficient of I + SN→path coefficient of A × A→path coefficient of I). For the influence of behavior attitude on behavior awareness, the influence coefficient is 0.689. Based on the theory of planned behavior, the influence mechanism model of the work safety behavior of MSEs in high-risk industries is constructed, as shown in Figure 3.

## Results

The following conclusions can be obtained through the above path analysis and mediation effect test:

(1) Work safety behavior attitude (A) positively affecting the behavioral intention (I) put forward by hypothesis 1 is verified, and its degree of influence is the highest. From the perspective of the path coefficient, the work safety behavior attitude most influences the behavioral intention (path coefficient is 0.689), which is higher than the direct influence of subjective norms or perceptual behavior control on the behavioral intention. It is proved theoretically that the behavior attitude of business owners is the main reason that directly affects the work safety behavioral intention of MSEs in high-risk industries (Mei et al., 2018). Changing the work safety behavior attitude of business owners is an important way to reduce work unsafe behaviors of MSEs.

(2) Work safety subjective norms (SN) positively affect the behavioral intention (I), and the work safety behavior attitude (A) plays a mediating role in the influence of work safety subjective norms (SN) on behavioral intention (I). From the perspective of the path coefficient, the subjective norm is significantly correlated with behavioral intention (path coefficient is 0.457). Combined with the mediating effect testing, the behavior attitude plays a mediating role in the influence of subjective norms on behavioral intention through safety attitude. The results show that subjective norm affects the behavioral intention directly through behavior attitude, and it can also positively affect the behavioral intention directly, with the largest path coefficient value and the highest influence degree (the influence coefficient is 0.81). Subjective norms embody the constraints by the external entities of the enterprise on work safety, which involves regulatory constraints from the government (hard norms), safety demands from corporate employees (Liu et al., 2019), public opinion pressure, the binding force of industry associations, and the work safety constraints of upstream and downstream enterprises in the supply chain (soft constraints). These factors present standard requirements for the work safety behavior and behavioral intentions of MSEs in high-risk industries.

(3) Work safety perceptual behavioral control (PBC) positively affects behavioral intention (I), and they are significantly correlated with each other (path coefficient is 0.411). Perceptual behavior control is an effective resource supply for work safety behavior. Validity analysis identifies three common factors, namely, internal resources of the enterprise (PBCCF1), support of service organizations (PBCCF2), and government support (PBCCF3). The internal resources of the enterprise include the management level of the enterprise, the safe operation capability of the employees, and the work safety software and hardware resources invested. The support of service organizations includes technical support such as work safety training, standardized guidance, and daily supervision provided to MSEs. Government support policies are the support resources provided by the government such as services and subsidies. Combined with the results of EFA, it is found that the eigenvalue and explained variance of the enterprise internal resources (PBCCF1) are significantly higher than that of support of service organizations and government support. Therefore, improving the effective resources, especially the internal resources of the enterprise, is of great significance in enhancing the behavioral intention of work safety.

(4) Work safety behavior habit (BH) is not significantly related with the behavioral intention (I). The test results are similar to the empirical research results by foreign scholars (research on the field of risk behavior), that is, the direct effect of behavior habits on behavioral intentions is not obvious. After the validity analysis, the behavior habits identify two common factors, the past behavior of the enterprise (BHCF1) and the code of conduct of neighboring enterprises (BHCF2). The past behavior of the enterprise reflects the past behavior formed in the long-term production, that is, the illegal operations that the enterprise will still implement as long as no accident occurs (Norman and Cooper, 2010). The code of conduct of neighboring enterprises reflects the work safety habits and investment in the safety of neighboring enterprises. Combined with the results of the EFA on behavioral habits, the eigenvalues and explanatory variances of past behaviors of enterprises are significantly higher than those of the code of conduct of neighboring enterprises. It can be seen that the illegal production behaviors formed over a long period of time directly affect their work safety behavior attitudes, thereby determining their behavioral intentions. The behavioral habit does not directly affect the behavioral intention, which can be reflected from the mediating role of the behavioral attitude in hypothesis 6.

(5) Work safety behavior attitude (A) plays a mediating role in the influence of behavior habit (BH) on behavioral intention (I). Combined with the conclusion of hypothesis 5, the behavior habit does not directly affect behavioral intentions (Ma et al., 2016) but influences behavioral intentions through the mediating effect of behavioral attitudes. The results show that behavior habits significantly and positively affect behavior attitudes (the path coefficient is 0.486). It implies that in the past, the convenience brought by some unsafe behaviors to the production of enterprises leads to the reduction of individuals’ awareness of the danger of unsafe production behaviors. The demonstration effect of work safety of neighboring enterprises also stimulates the investment in work safety of the enterprise, greatly affecting its work safety behavior. It is easier to change business owners’ behavior attitudes through the standardization of the behavioral habits of MSEs in high-risk industries, especially their past behaviors, and their safety awareness can be enhanced.

(6) Work safety risk consciousness (RC) significantly influences the behavioral intention (I), but the influence degree is the lowest (the path coefficient is –0.289). Risk consciousness is negatively correlated with behavioral intention. This result shows that enterprises with higher risk preferences are more likely to conduct unsafe production behaviors. The risk consciousness variable is taken as an exogenous variable supplemented in TPB theory. The path coefficient shows that it has the weakest influence on the behavioral intention of work safety. However, the introduction of it can improve the explanatory power of the work safety behavior model of MSEs in high-risk industries to a certain extent, and the problem is explained more comprehensively and sufficiently. In the research on the work safety behavior of MSEs in the high-risk industries, risk-conscious behaviors are more likely to form unsafe behavioral intentions (Tucker and Turner, 2013), causing work unsafe behaviors. It is suitable for this variable to be a supplement to TPB theory.

(7) Work safety behavioral intention (I) significantly influences the safety behavior (B), and they are significantly correlated with each other (the path coefficient is 0.457). After the validity analysis, three common factors are identified by safety behaviors, namely, safety management (BCF1), safety training (BCF2), and safety prevention (BCF3). These three aspects are well developed in work safety behavior and are recognized by scholars (Lu et al., 2016). Combined with EFA of behavior, it is found that the eigenvalues and explanatory variances of the three common factors are relatively close, and the safety training value is slightly higher. It shows that the behavioral intention of work safety affects the safety management, safety training, and safety prevention of the enterprise. The behavioral intention of MSE’s owners influences the work safety behaviors of MSEs through the decision of the business owner to realize the work safety behavior of MSEs.

## Discussion

### Theoretical Significance

Considering the risk characteristics of MSEs in high-risk industries, we study the work safety attitude of MSE’s owners with decision-making power, and then we expand the traditional TPB model. Under the joint action of MSEs in high-risk industries, government safety supervision department, work safety service agencies, and related subjects of social sanction, it is studied the different factors affect the work safety behavior of MSEs. It reduces the influence mechanism of work safety behavior of MSEs in high-risk industries and further identifies the key factors affecting the formation of work safety behavior.

(1) From the perspective of MSEs, there are many factors for the work safety behavior of MSEs in high-risk industries. The key factors are the work safety attitude of the business owner, the enterprise work safety resources, the risk consciousness, and the past behavior of the enterprise. These factors determine the behavioral intention and further determine the work safety behavior. According to the influence degree, the most important factor is the work safety attitude of the business owner, followed by the enterprise work safety resources.

(2) From the perspective of the government safety supervision department, it is studied the influence of daily supervision, penalties for violations, accountability, and penalty for accidents, purchasing the services of the qualified enterprises, providing subsidies and rewards by the government on enterprise behavioral intentions. The key factors are government supervision, government punishment, and government subsidies.

(3) From the perspective of work safety service agencies, it is explored the influence of the technical support and supervision and management provided for MSEs that purchase entrusted business by service organizations on behavioral intentions. It can be seen that work safety service level (focus on technology and supervision) is the key factor.

(4) From the perspective of social sanction, it involves the work safety constraints of public opinion, industry associations, and upstream and downstream enterprises in the supply chain of MSEs. The influence of neighboring enterprises on the behavior habits of small and micro enterprises is considered. The factors influence behavioral intentions, and different enterprise behaviors are formed. The key factors are social constraints and influence of neighbor enterprise behavior.

**FIGURE 4:**
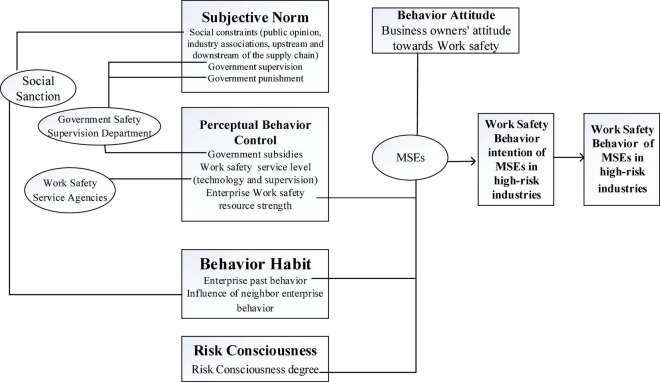
Influence mechanism of work safety behaviors of MSEs in high-risk industries.

Hence, the influence mechanism of work safety behaviors of MSEs in high-risk industries is obtained, as shown in Figure 4.

### Practical Significance

The influencing mechanism and path of work safety behavior of MSEs in high-risk industries are analyzed, and the key influencing factors of work safety behavior of MSEs are revealed. It is further explored how to improve the awareness of work safety, stimulate the internal drive of work safety, and guide the transformation of work safety behavior of MSEs from a negative response to positive pursuit.

(1) It is a fundamental way to reduce the work unsafe behavior of MSEs by changing the behavior attitude of the enterprise owners, which directly affects the intention of work safety behavior.
(2) It is a basic guarantee to realize the work safety behavior of MSEs by effective resource supply for work safety behavior of MSEs. It positively affects the intention of work safety behavior from three dimensions, namely, internal strength resources, service organization support resources, and government support resources. In particular, the internal strength resources of enterprises play a major role in the promotion of work safety behavior.
(3) The behavior habits in high-risk industries formed by enterprises for a long time has a direct effect on the behavior attitude of enterprises toward work safety, which acts on the behavioral intentions through the intermediary function of behavior attitude. Enterprises with strong risk preferences are more likely to have the tendency of work unsafety behavior.
(4) Subjective norms mainly come from the government safety supervision departments, work safety service agencies, and social sanctions. Subjective norms have a direct impact on behavioral intention and play an intermediary role. Its overall impaction is the greatest.

It can be seen that the four key factors affecting the work safety behavior of MSEs in high-risk industries are the strength of enterprise work safety resources, the supervision of the government safety supervision department, the degree of government service subsidies, and the service level of work safety service agencies.

### Limitations and Future Work

When conducting empirical analysis based on the theory of planned behavior, it is necessary to conduct a questionnaire survey on the business owners of MSEs in high-risk industries in China. Affected by the epidemic, it is only investigated the provinces in the east of China, involving MSEs’ owners in high-risk industries such as machinery manufacturing, constructional engineering, production and storage of hazardous material, transportation, non-coal mining, fireworks production, and metallurgy. There is little research on provinces with relatively backward economic development in China. In addition, the exploitation of small coal mines has been banned as early as 2005 in some provinces in the east of China (such as Jiangsu Province). Therefore, the interviewees and questionnaires in this study do not involve industries in areas with a high incidence of work safety accidents and the hardest-hit areas. The study on high-risk industries such as coal production, research, production, and testing of weapons and equipment (including civil aviation and nuclear fuel) has a positive guiding role and practical significance for enriching and enhancing the work safety behavior of MSEs. We will conduct an in-depth research on it when conditions and resources are sufficient in the future.

## Conclusion

It is pointed out that “the lucky psychology + the limited resource” is the root of the passive work safety behavior of MSEs in high-risk industries. The work safety behavior of MSEs is affected by the joint action from MSEs, government safety supervision departments, work safety service agencies, and social sanctions. We should strengthen the government supervision, punishment, and subsidies; seek the service support of work safety service agencies; and enlarge the control of constraints from society. Only in this way, the enterprises could reduce the work unsafety behavior of MSEs as far as possible. We should stimulate the internal driving force of work safety behavior and ensure the safe production capacity of MSEs, which will improve the overall work safety level of MSEs in high-risk industries.

## Data Availability Statement

The original contributions presented in the study are included in the article/Supplementary Material, further inquiries can be directed to the corresponding author/s.

## Ethics Statement

The studies involving human participants were reviewed and approved by the Ethical Review Committee of Jiangsu University in China. The patients/participants provided their written informed consent to participate in this study.

## Author Contributions

WL and SL investigated the data and carried out the project administration. XN and XZ carried out the data curation. WL and XN carried out the formal analysis. QM carried out the funding acquisition and supervised the data. WL performed the methodology, carried out the resources, and wrote the manuscript. XN validated the data. All authors have read and agreed to the published version of the manuscript.

## Conflict of Interest

The authors declare that the research was conducted in the absence of any commercial or financial relationships that could be construed as a potential conflict of interest.

## Publisher’s Note

All claims expressed in this article are solely those of the authors and do not necessarily represent those of their affiliated organizations, or those of the publisher, the editors and the reviewers. Any product that may be evaluated in this article, or claim that may be made by its manufacturer, is not guaranteed or endorsed by the publisher.
