# Supplementary material for: Study on Influencing Factors of Micro and Small Enterprises’ Work Safety Behavior in Chinese High-Risk Industries
Source: Front Psychol. 2022 May 16;13:880205. doi: 10.3389/fpsyg.2022.880205 (PMC9150854; doi:10.3389/fpsyg.2022.880205)
Supplement: Supplementary file 1 [file Table_10.docx]

**Appendix**

A formal questionnaire of MSEs’ work safety behavior in high-risk industries is shown in Table 10.

**TABLE 10 |** The formal questionnaire of MSEs’ work safety behavior in high-risk industries.

| **Dimension** | **No** | **Item** | **References** |
| --- | --- | --- | --- |
| Behavior  Attitude  (A) | A1 | Work safety is an important part of the operation strategy, and the work safety goal is clearly defined in the enterprise strategic planning. | Ajzen (991,2011),  Cordano & Frieze(2000)，  Zwanikken(2011),  Liu(2012) |
|  | A2 | Work safety is an important part of enterprise operation management and plays an important role in the production |  |
|  | A3 | Even if the probability of work safety accidents is small, it still needs attention |  |
|  | A4 | Enterprises are willing to increase investment in work safety to ensure production safety |  |
|  | A5 | Enterprises increase investment in work safety can improve safety economic benefits |  |
|  | A6 | When there are work safety problems, the enterprise can take the initiative to report to the relevant departments |  |
|  | A7 | Enterprises are willing to participate in work safety and eliminate potential safety hazards in time |  |
| Subjective  Norms  (SN) | SN1 | The government continues to strengthen laws and regulations on work safety to the constraint force | Ajzen(1991,2011),  Southey (2011),  Oliver& Bearden(2008),  Wu et al. (2011) |
|  | SN2 | The local work safety supervision department of the government regularly conducts effective work safety inspections on enterprises to help them analyze the work safety situation |  |
|  | SN3 | Effective hazard rectification and tracking procedures are established t o cope with potential dangers of the enterprise, and the production will not be allowed until the hidden dangers are eliminated |  |
|  | SN4 | According to the industry characteristics, employees put forward standardized requirements for production conditions |  |
|  | SN5 | For dangerous positions, employees take the initiative to put forward training requirements for standardized operations |  |
|  | SN6 | The enterprise actively implements the rectification suggestions put forward by the service organization |  |
|  | SN7 | The pressure of public opinion from the news media promotes the standardization of work safety behavior of enterprises |  |
|  | SN8 | The public right to know in the community enhances the awareness of work safety of the enterprise |  |
|  | SN9 | Industry norms increase the binding force of the enterprise’s work safety behavior |  |
|  | SN10 | Large customers greatly restrict the work safety and safety protection of enterprises |  |
|  | SN11 | Suppliers of raw materials and accessories strengthen the enterprise’s work safety environment requirements |  |
| Perceptual  Control  Behavior  (PBC) | PBC1 | When implementing work safety standardization evaluation, the enterprise is able to improve the status quo of work safety | Ajzen(1991,2011)，  Cordano & Frieze (2000)  Zhang (2008) ,  Chen (2011) |
|  | PBC2 | Enterprises are fully capable of understanding safety information such as safety laws and regulations, safety technical standards, work safety encouragement policies, etc. |  |
|  | PBC3 | The low education level and weak safety awareness of employees greatly affect the implementation of work safety standardization |  |
|  | PBC4 | Weak safety operation ability of grassroots employees greatly restricts the work safety level of the enterprise |  |
|  | PBC5 | Enterprises can easily obtain work safety services from service organizations, such as training methods, technical services standardization, etc. |  |
|  | PBC6 | Safety service agencies carry out work on enterprises in strict accordance with the requirements of laws |  |
|  | PBC7 | The safety training, education, evaluation, and acceptance of the service organization are conducive to the service organization’s effectiveness in supervising the enterprise’s work safety |  |
|  | PBC8 | The safety supervision departments in local government take the initiative to provide work safety service measures |  |
|  | PBC9 | The safety supervision departments in local government provide effective policies to help enterprises implement standards |  |
| Behavior  Habit  (BH) | BH1 | In the past, enterprises devoted themselves to establishing and supervising the work safety regulations | Bagozzi&Kimmel(1988），  Ajzen(1991,2011)，  East(1993),  Podu(2008) |
|  | BH2 | The enterprise used to supervise and rectify their main sources of danger in a timely manner |  |
|  | BH3 | The enterprise has been committed to building a work safety management organization and appointing full-time safety officers |  |
|  | BH4 | The equipment and production environment are regularly inspected, and keep recording and tracking |  |
|  | BH5 | The enterprise has always attached importance to pre-job training for new or rotation employees |  |
|  | BH6 | The enterprise has been offering cash rewards to employees for voluntarily reporting potential safety hazards |  |
|  | BH7 | The enterprise has always attached importance to the renewal and maintenance of production equipment, and increasing the intensity of technical reform |  |
|  | BH8 | The enterprise implements safety investment within the scope of national regulations, with regulated and legal investment intensity |  |
|  | BH9 | The demonstration effect of the work safety investment of neighboring enterprises greatly stimulates the work safety investment of the enterprise |  |
|  | BH10 | The work production behavior of neighboring enterprises greatly affects the work safety behavior of the enterprise |  |
| Risk Consciousness  (RC) | RC1 | The enterprise has set up obvious warning signs for existing hazards | Purswell et al.(1997)，  Ortiz et al.(2009)，  Wang(2011) |
|  | RC2 | For major hazards, the enterprise is willing to standardize operating procedures and actively implement continuous training for employees |  |
|  | RP3 | No personal injury or economic loss, and the enterprise wants to take the plunge |  |
|  | RC4 | The hazards of safety accidents are more important than other issues involved in the production and operation of the enterprise |  |
|  | RC5 | Any minor damage will issue a warning for next series of safety accidents |  |
|  | RC6 | The risks in work appear at specific times and places. As these times and are staggered, it is feasible to operate in violation of regulations occasionally. |  |
| Behavioral  Intention  (I) | I1 | The enterprise is willing to increase safety spending in the coming year | Ajzen(1991,2011)，  East(1993)，  Emilio (2002) |
|  | I2 | The enterprise is willing to incorporate work safety into the mid- and long-term plans for future development |  |
|  | I3 | In the case of fund shortage, the enterprise will greatly reduce the human, money, and material invested in safety |  |
|  | I4 | The enterprise is willing to purchase injury insurance for employees |  |
|  | I5 | The enterprise is willing to ensure that the injured employees get the corresponding economic compensation and guarantee their living standards |  |
| Work  Safety Behavior  (B) | B1 | The enterprise improves the regulations of work safety, and ensures that all departments and employees of the enterprise understand them | Ajzen(1991,2011），  Neal et al.(2000），  Lin Suina (2005)  Beatriz(2006)，  Evans(2008),  Vinodkumar (2010)，  Johnstone (2011),  Leka (2011),  Hale (2013) |
|  | B2 | The enterprise clearly understands the relevant safety policies and work safety laws and regulations of the government supervision department |  |
|  | B3 | The enterprise sets perfect work safety management organization with full-time (or part-time) safety management personnel |  |
|  | B4 | Continuously improve the work safety responsibility system, and perform regular assessments and rectifications |  |
|  | B5 | Business owners actively participate in work safety training to improve their awareness of work safety |  |
|  | B6 | The enterprise provides pre-job safety education and training for new employees |  |
|  | B7 | The enterprise provides on-the-job employees with comprehensive training on work safety and health regularly |  |
|  | B8 | The enterprise conducts regular safety education and training on possible safety emergencies |  |
|  | B9 | The enterprises arrange physical examination for employees regularly, and increases the number of physical examinations for employees in dangerous positions |  |
|  | B10 | Monitor the important positions with potential hazards and make reports in time |  |
|  | B11 | Continuously promote enterprise technological transformation and equipment renewal to enhance the work safety capacity |  |
|  | B12 | Enterprises take work safety expenses in accordance with national regulations to ensure that the special funds are used exclusively |  |
|  | B13 | The enterprise cope with the harmful factors in the production environment and set up safety protection for the equipment with potential hazards |  |
|  | B14 | Necessary protective equipment is provided for all employees. Inspect and maintain safety protective measures and protective equipment regularly. |  |
|  | B15 | The enterprise sets up emergency exists and resources to save the lives of employees in time |  |
